# Comparison of body composition, cardiorespiratory, and neuromuscular adaptations induced by three different high intensity training protocols

**DOI:** 10.14814/phy2.70306

**Published:** 2025-04-01

**Authors:** Corentin Scoubeau, Julie Carpentier, Stéphane Baudry, Vitalie Faoro, Malgorzata Klass

**Affiliations:** ^1^ Cardio‐Pulmonary Exercise Laboratory Faculty of Human Motor Sciences, Université Libre de Bruxelles Brussels Belgium; ^2^ Research Unit in Biometry and Exercise Nutrition Faculty of Human Motor Sciences, Université Libre de Bruxelles Brussels Belgium; ^3^ Laboratory of Applied Biology and Research Unit in Applied Neurophysiology Faculty of Human Motor Sciences, ULB Neuroscience Institute, Université Libre de Bruxelles Brussels Belgium

**Keywords:** health‐related fitness, muscle endurance, perceived exertion, strength, VO_2_peak, voluntary activation

## Abstract

This study investigated body composition, cardiorespiratory, and neuromuscular adaptations induced by three high intensity trainings easy to fit into daily routine. Thirty‐seven adults participated in one of the following 8‐week interventions: vigorous intensity continuous training (VICT; 28 min at 70% of peak oxygen uptake [VO_2_peak]), long interval high intensity interval training (LI‐HIIT; 6 × 2 min at 85% VO_2_peak), or short interval HIIT (SI‐HIIT; 12 × 30 s at 125% maximal power output). Heart rate (HR) and rating of perceived exertion (RPE) were measured during sessions. Pre‐ and post‐intervention assessments included fat and lean mass, cardiopulmonary exercise testing, knee extensors maximal isometric torque, voluntary activation, and endurance during a submaximal contraction. Compared to SI‐HIIT and VICT, LI‐HIIT sessions were characterized by a shorter duration, a similar time spent above 90% HR_max_, but a higher RPE (*p* < 0.05). VO_2_peak and muscle endurance increased respectively by 14% and 12%, while knee extensors torque, voluntary activation, and lean mass increased to a lesser extent (1%–3%) after the interventions (ANOVA time‐effect, all *p* < 0.05). There was no significant difference between the modalities (intervention × time interaction, all *p* > 0.05). In conclusion, comparable body composition, cardiorespiratory, and neuromuscular adaptations were induced by the three high intensity training protocols, while RPE was higher during LI‐HIIT sessions.

## 1 INTRODUCTION

Higher physical activity and fitness levels bring many health benefits and prevent cardiometabolic diseases (Blair et al., 2001; Reiner et al., 2013). Unfortunately, one third of the population fails to meet the physical activity recommendations established by the World Health Organization (WHO, 2024a).

To overcome the most cited barrier to engaging in physical activity, namely the lack of time (Koh et al., 2022), and induce a faster increase in physical fitness, higher intensity training has been proposed as a time‐efficient alternative to moderate intensity continuous training (MICT) in healthy adults and patients (Burgomaster et al., 2008; Hannan et al., 2018; Herrod et al., 2021; Maillard et al., 2016; Sultana et al., 2019; Weston et al., 2014). Two main forms of high intensity training exist: high intensity interval training (HIIT) and vigorous intensity continuous training (VICT).

HIIT consists of either long intervals (usually 2–4 min; LI‐HIIT), generally performed at vigorous to near maximal intensities (American College of Sports Medicine, 2018), or short intervals (6–60 s) (MacInnis & Gibala, 2017; Weston et al., 2014). The latter are often mentioned as sprint interval training (SIT) and refer to either repeating Wingate tests or short bouts of all‐out exercise (Sloth et al., 2013). However, this type of effort may be barely achievable and poorly tolerated in the general population. Therefore, new forms of short interval HIIT (SI‐HIIT) have been developed with intensities more adapted for inactive or moderately active subjects (Astorino et al., 2013; Bayati et al., 2011; de Oliveira‐Nunes et al., 2021). VICT is another form of high intensity aerobic training that was primarily proposed for healthy individuals (Islam et al., 2019; Myrkos et al., 2023), but also for patients with breast cancer and overweight subjects (Kong et al., 2016; Li et al., 2021; Maginador et al., 2020). VICT was shown to induce greater improvements in peak oxygen uptake (VO_2_peak) than MICT of equivalent volume and could be a more time‐efficient modality than MICT as session duration was shorter for VICT (Gormley et al., 2008).

Exertion perceived during exercise was identified as one of the factors predicting in‐task affective valence (Farias‐Junior et al., 2020) which could impact adoption and adherence to exercise, mainly in inactive subjects (Biddle & Batterham, 2015; Williams, 2008). Rating of perceived exertion (RPE) was measured during single sessions of SI‐HIIT, LI‐HIIT, and MICT, and a lower RPE was reported during MICT and SI‐HIIT compared to LI‐HIIT (Naves et al., 2019). Some have raised concern that VICT may be too strenuous for individuals less familiar with physical activity, making interval training a more feasible alternative (Jung et al., 2014; Maginador et al., 2020). However, to the best of our knowledge, RPE during VICT was not yet compared to SI‐HIIT and LI‐HIIT during interventions of several weeks. In addition, although there is abundant literature comparing the effectiveness of HIIT and MICT on the main health‐related fitness components (i.e., cardiorespiratory fitness, body composition, muscle strength, and endurance) (Gibala et al., 2012; Hwang et al., 2019; Sultana et al., 2019), no study directly compared the effects of SI‐HIIT, LI‐HIIT, and VICT.

To improve health‐related fitness and facilitate physical activity participation in people lacking time, it appears important to identify the most tolerable (i.e., inducing lower RPE) and efficient exercise interventions, including different HIIT regimes and VICT. Therefore, this study aims to compare the effects of VICT, SI‐HIIT, and LI‐HIIT over 8 weeks on the main components of health‐related fitness, but also the physiological responses (i.e., heart rate and time spent in different intensity zones) and perceived exertion during training sessions. We hypothesized that the time spent at vigorous to near‐maximal intensity attained during the 3 modalities will induce comparable cardiorespiratory adaptations, while neuromuscular adaptations could differ due to different metabolic disturbances and neuromuscular load induced by the 3 modalities (Buchheit & Laursen, 2013a, 2013b). Regarding RPE during sessions, we expected it to be lower during SI‐HIIT, compared to LI‐HIIT and VICT, due to the shorter bouts of exercise at high intensity.

## 2 MATERIALS AND METHODS

### 2.1 Participants

The protocol of the study was approved by the Erasmus hospital (Brussels, Belgium) Ethical Committee (reference: B406201836213). All the participants received oral and written information regarding the protocol and signed a written informed consent.

**FIGURE 1:**
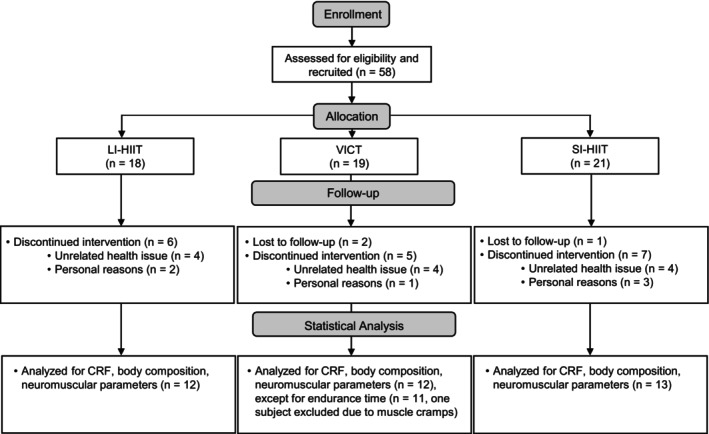
Study flowchart. CRF, cardiorespiratory fitness; LI‐HIIT, long interval high intensity interval training; SI‐HIIT, short interval high intensity interval training; VICT, vigorous intensity continuous training.

Participants were recruited through local advertisement, social media, and by word of mouth on the university campus. To be included, participants needed to be between 18 and 50 years old, inactive to moderately active at most (i.e., below or within the World Health Organization physical activity recommendations; 150–300 min of moderate intensity, 75–150 min of vigorous intensity physical activity, or an equivalent combination of both) (WHO, 2024b), and not be involved in any structured endurance or strength training program in the last 6 months. Individuals with a condition limiting participation in maximal physical tests and training were excluded. Before inclusion, participants completed the global physical activity questionnaire (WHO, 2024c) to ensure they met inclusion criteria regarding physical activity participation. After inclusion, participants were randomly assigned to one of the 3 exercise interventions, namely LI‐HIIT, SI‐HIIT, or VICT, using a stratified randomization by sex (two strata: male and female). Initially, 58 participants were included in the study, but 18 discontinued the intervention, mostly due to a health issue unrelated to their participation in the study (*n* = 12) or schedule incompatibilities (*n* = 6), and 3 were lost to follow‐up. The final sample comprised 12 participants in the LI‐HIIT group (7 females, 24 ± 5 years), 13 in the SI‐HIIT group (7 females, 23 ± 3 years), and 12 in the VICT group (6 females, 24 ± 3 years). One participant from the VICT group had to be excluded from the analysis of endurance time due to muscle cramps during the post‐intervention session. A flowchart is presented in Figure 1 and participants' characteristics (male/female ratio, age, and BMI) and participation in vigorous and moderate physical activities, based on the global physical activity questionnaire filled in at inclusion, are presented in Table 1.

**TABLE 1:** Characteristics and recreational physical activity of the subjects at inclusion.

Group	Ratio ♂/♀	Age (yrs)	BMI (kg/m^2^)	Vigorous PA (min/week)	Moderate PA (min/week)
VICT	6/6	23.5 ± 2.7	22.9 ± 3.5	0 [0, 30]	10 [0, 112]
LI‐HIIT	5/7	24.1 ± 4.9	26.3 ± 5.0^a^	0 [0, 90]	45 [0, 105]
SI‐HIIT	6/7	22.6 ± 2.8	21.9 ± 2.0	0 [0, 105]	90 [0, 135]

*Note*: Data presented as mean ± standard deviation or median [25th, 75th percentile] depending on distribution. Participation in vigorous and moderate intensity physical activities did not differ between groups (Kruskal–Wallis test, *p* = 0.527).

Abbreviations: BMI, body mass index; LI‐HIIT, long interval high intensity interval training; PA, physical activity; SI‐HIIT, short interval high intensity interval training; VICT, vigorous intensity continuous training.

^a^ BMI was slightly higher in the LI‐HIIT group compared to the SI‐HIIT group (post‐hoc test, *p* < 0.05).

### 2.2 Study design

**FIGURE 2:**
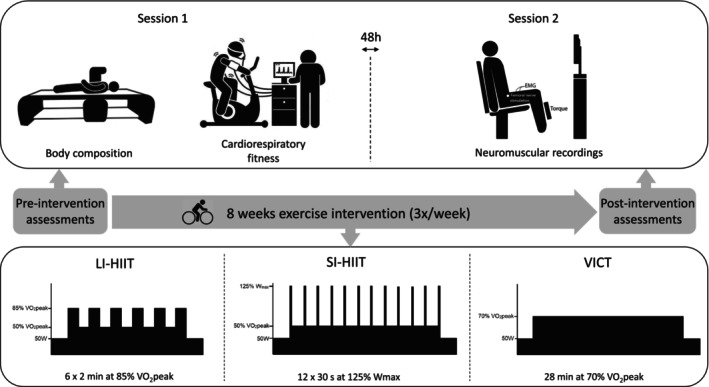
Study design. EMG, Electromyographic recording; LI‐HIIT, long interval high intensity interval training; SI‐HIIT, short interval high intensity interval training; VO_2_peak, peak oxygen uptake; VICT, vigorous intensity continuous training; W_max_, maximal power output.

The study design is represented in Figure 2. Before and after the exercise interventions, evaluations were conducted over 2 sessions interspaced by 48 h. The first session was dedicated to the assessment of body composition and cardiorespiratory fitness through classical cardiopulmonary exercise testing (CPET). The second session was dedicated to the recording of neuromuscular parameters.

### 2.3 Body composition assessment

After an overnight fast, total and segmental body composition was measured through Dual‐energy X‐ray absorptiometry (DXA, Lunar Prodigy, GE Healthcare, USA) and analyzed using the enCORE software (version 15.0) (Toombs et al., 2012).

### 2.4 Cardiopulmonary exercise testing

Subjects took part in an incremental CPET on an electrically braked stationary bike (Ergoselect 100, Ergoline GmbH, Germany) according to standard recommendations (Wasserman et al., 1973). The exercise protocol included a 3‐min warm‐up phase at 20 W for females and 30 W for males, followed by workload increments of 15 W/min for females and 20 W/min for males until volitional exhaustion. Throughout the test, measures of oxygen uptake (VO_2_), ventilation, and carbon dioxide production were collected breath by breath using a tightly fitted oro‐nasal mask connected to a cardiopulmonary exercise system (Ergocard, Medisoft, Belgium) calibrated with room and standardized gas. Heart rate (HR) was monitored through a 12‐lead EKG. The test was considered maximal when two of the following criteria were met: [1] the participant reached a plateau of VO_2_ (less than 100 mL/min of increase in VO_2_ with a further increase in workload), [2] a respiratory exchange ratio over 1.15, [3] achievement of age‐predicted HR_max_ (220 – age), [4] inability to maintain pedaling frequency above 50 rpm. Since a plateau of VO_2_ was not observed in most subjects, the VO_2_peak was taken as the highest level of VO_2_ reached during the last fully achieved stage, and maximal power output (W_max_) was the corresponding workload. The first ventilatory threshold (VT1) was determined using the V‐slope and the ventilatory equivalent method (Reinhard et al., 1979; Wasserman et al., 1973) by two independent investigators.

### 2.5 Neuromuscular assessment

For the neuromuscular recordings, subjects were sitting on an adjustable chair with their back supported by the chair and with the hip and knee angles at 100°. A force transducer (linear range, 0–2500 N; U2000 load cell, Maywood Instruments Ltd., Basingstoke, UK) was fixed to the front of the chair and attached to the right leg by a Velcro strap (2 cm above the lateral malleolus) to measure the isometric torque produced by the knee extensors (Klass et al., 2016). Electromyographic activities (EMG) were recorded from the rectus femoris, vastus medialis, vastus lateralis, and biceps femoris using self‐adhesive electrodes (Red Dot, 3 M, Minnesota, USA). To guarantee comparable recording circumstances during pre‐ and post‐intervention sessions, the placement of the EMG electrodes was recorded during the first session. EMG signals were amplified (×1000) and filtered (10 Hz – 1 kHz) by a custom‐made differential amplifier. Electrical stimulation delivered to the femoral nerve by a constant current stimulator (DS7AH Digitimer, Welwyn Garden City, UK; pulse duration 200‐μs) and self‐adhesive electrodes was used to assess voluntary activation and muscle contractile properties. The precise location of the stimulation was determined at rest using weak electrical stimulation. Stimulation intensity was then gradually increased to attain maximal twitch torque and set 30% above that intensity to ensure maximal muscle activation. Torque and EMG signals were acquired on a computer at a sampling rate of 2 kHz with a data‐acquisition system (Model MP 150, Biopac Systems, Santa Barbara, CA, USA) and analyzed off‐line with associated AcqKnowledge 4.1 software.

During the recording sessions, participants performed five 3‐s isometric maximal voluntary contractions (MVC), interspaced by a 3‐min rest interval; the first two as familiarization, and the last 3 for recordings. MVC torque and associated average value of the rectified EMG (aEMG) of vastus medialis, vastus lateralis, rectus femoris, and biceps femoris were determined for a 500‐ms period during the plateau of the 3 MVC. Values obtained from the two attempts that produced the highest MVC torque were averaged.

Voluntary activation was assessed using the interpolated twitch technique with paired supramaximal electrical stimuli delivered at 10‐ms intervals during the MVC plateau and immediately after the MVC (Klass et al., 2016). The superimposed torque was defined as the difference between the superimposed peak torque and the MVC torque prior to the stimulation, while the resting twitch torque was defined as the difference between the peak twitch torque and the baseline. Voluntary activation level was calculated according to the following equation: (1 – superimposed torque/resting twitch torque) × 100 (Shield & Zhou, 2004).

Finally, muscle endurance (i.e., the capacity to sustain a given percentage of MVC torque over time) (American College of Sports Medicine, 2018) was measured after a 10‐min rest period. Participants had to maintain a contraction equivalent to 30% MVC for as long as possible with a visual feedback of the target torque (Martinez‐Valdes et al., 2017). Endurance time was measured from the beginning of the contraction till the moment the subject's torque decreased by 10% below the target torque for at least 5 s.

### 2.6 Exercise interventions

The 3 interventions lasted 8 weeks at a frequency of 3 sessions per week. Participants were all working or studying on the campus and performed the training sessions on stationary bikes accessible during the opening hours of our building. They were requested and reminded several times to keep their dietary and other physical activity habits unchanged during the intervention. For prescribing exercise intensity, various methods exist. Domain‐based exercise intensity prescription (Inglis et al., 2024) or determining intensities based on the critical power (Lipková et al., 2022) better accounts for individual differences in metabolic and physiological responses to exercise. However, these methods require several testing sessions, which are difficult to implement in daily practice, particularly for busy inactive to moderately active individuals. Given our objective to align exercise prescription with real‐world feasibility, we opted to determine training loads as a percentage of VO_2_peak. This widely used approach provides a practical and accessible means of prescribing exercise intensity while still ensuring a physiologically relevant stimulus (Taylor et al., 2019).

The stationary bikes were connected to the Ergoline Rehab System (ERS.2 software, version 1.07, Ergoline GmbH, Germany). This system allows automatic and instantaneous switching between pre‐encoded training workloads based on participants' performance during CPET. All protocols started with a 3‐min warm‐up at a load of 50 to 75 W and ended with a 2‐min cool down. The VICT protocol consisted of cycling 28 min at a load corresponding to 70% of VO_2_peak, LI‐HIIT consisted of 6 bouts of 2 min at a load corresponding to 85% of VO_2_peak, and SI‐HIIT consisted of 12 bouts of 30 s at 125% of W_max_. For both modalities, exercise intervals were interspaced by 2‐min recovery periods at a load equivalent to 50% of VO_2_peak. The conditioning phase of the LI‐HIIT and SI‐HIIT lasted, respectively, 22 and 28 min. For all modalities, training intensity was increased every week by 2% of the load corresponding to VO_2_peak (W_max_). A progression in percentage of W_max_ was previously used in studies comparing different HIIT regimes (Astorino et al., 2013). It was chosen because it was easy to implement, was applicable to the 3 modalities, and avoided calculations based on VO_2_ measured during the CPET and changes to the number of intervals or session duration.

Protocols of the 3 interventions were designed to be achievable by the target population and to fit in a ~30 min time‐window. This timeframe was indeed deemed by participants as compatible with their daily routine activities. Protocols were determined during preliminary sessions carried out on 6 inactive subjects to ensure their applicability. During those sessions, based on the interval duration and target intensities fixed for the two HIIT protocols (see above), the number of intervals achievable during a ~30‐min session of LI‐HIIT and SI‐HIIT was determined. The LI‐HIIT session was the most strenuous, and 4 out of the 6 subjects could not repeat more than 6 intervals, leading to the decision to limit the number of intervals per session to 6. This was decided to avoid that part of the subjects do not complete the exercise session as it happened in previous studies (Oliveira et al., 2013). Regarding SI‐HIIT, all the subjects were able to perform 12 intervals. VICT duration was aligned with the SI‐HIIT protocol, and we then confirmed the 6 subjects were able to achieve the session.

Since training intensity influences exercise‐induced adaptations (Buchheit & Laursen, 2013a; MacInnis & Gibala, 2017), HR was continuously recorded during sessions using HR sensors provided with the Ergoline Rehab System. For the conditioning phase of each session, mean and peak HR were extracted from the ERS.2 software, and the time spent in different intensity zones, expressed as a percentage of the HR_max_ measured during the CPET (i.e., 60%–70%, 70%–80%, 80%–90%, and >90% HR_max_), was calculated. Immediately after the end of the exercise, participants noted the RPE during the session using the modified 0–10 Borg scale (Borg, 1982).

### 2.7 Statistical analysis

Normality of the data was controlled using a Shapiro–Wilk normality test. Since initial characteristics may influence adaptations to exercise, depending on the normality of the distribution, a one‐way ANOVA or a Kruskal–Wallis test was conducted to control for differences in participation in vigorous and moderate physical activities, body composition, cardiorespiratory, and neuromuscular variables between groups at baseline. The same statistical tests were used to compare the RPE, mean and peak HR data averaged over all sessions, and the time spent in each intensity zone during the 3 interventions. When a significant main effect was found, Dunn's or Bonferroni's post hoc tests were performed.

When normal distribution of data was verified, a two‐factor ANOVA (time [pre‐ vs. post‐intervention] × intervention [LI‐HIIT vs. SI‐HIIT vs. VICT]) with repeated measures on time was used to analyze training induced changes. Effect sizes were reported using partial eta‐squared (partial *η* ^2^). Values of 0.01, 0.06, and 0.14 correspond respectively to small, medium, and large effects (Lakens, 2013). For one variable presenting a non‐normal distribution (i.e., trunk fat mass), within‐group changes were tested using the Wilcoxon test. Statistical analysis was conducted with Jamovi (version 2.2.5). Considering our final total sample size (*n* = 37), divided into 3 groups (VICT, SI‐HIIT, LI‐HIIT) and 2 repeated measurements in each group, with an alpha level of 0.05, and assuming a minimal correlation of 0.7 among the repeated measures based on a previous study (Scoubeau et al., 2023), our design was sensitive to detect a moderate effect size (*f* = 0.25) with a power of 0.9 for the interaction between the within and the between group factors (Gpower version 3.1.9.6) (Faul et al., 2007; Scoubeau et al., 2022).

To assess the contribution of neural changes to the improvement in MVC torque, we correlated the percentage of change in voluntary activation and aEMG of the agonist muscles to the change in MVC torque between baseline and post‐intervention. In addition, we correlated baseline levels of voluntary activation and their percentage of change after the interventions to verify if the effect of the interventions could be higher in subjects with initially lower motor output. Depending on the distribution, Pearson (*r*_p_) or Spearman (*r*_s_) correlation coefficients were calculated.

## 3 RESULTS

### 3.1 Attendance rate, HR, time spent in different intensity zones, and RPE during training sessions

Among participants who completed the interventions, 5 participants (3 in VICT, 1 in SI‐HIIT, 1 in LI‐HIIT) missed 1 session, 2 participants (1 in VICT, 1 in SI‐HIIT) missed 2 sessions, and 1 participant (in LI‐HIIT) missed 4 sessions. Mean attendance was above 98% in all 3 groups.

Table 2 presents the time spent in the different intensity zones during the 3 interventions. The Kruskal–Wallis test identified significant differences between interventions for the 70%–80% HR_max_ (*p* = 0.040) and the 80%–90% HR_max_ (*p* = 0.035) zones. The post hoc tests indicated that the time spent between 80% and 90% HR_max_ was significantly lower during LI‐HIIT compared to VICT (*p* = 0.030).

**TABLE 2:** Time spent in the different intensity zones.

Intensity zone	Time spent (min:Sec)	Kruskal–Wallis *p*‐value
60%–70% HR_max_		
VICT	0:37 [0:27, 0:44]	0.604
LI‐HIIT	0:31 [0:22, 0:55]	
SI‐HIIT	0:23 [0:10, 1:08]	
70%–80% HR_max_		
VICT	2:54 [1:07, 4:15]	**0.040**
LI‐HIIT	5:06 [4:11, 6:57]	
SI‐HIIT	6:51 [1:39, 12:27]	
80%–90% HR_max_		
VICT	19:30 [9:45, 20:58]	**0.035**
LI‐HIIT	11:06 [10:13, 12:31]^a^	
SI‐HIIT	14:11 [8:27, 15:40]	
>90% HR_max_		
VICT	4:39 [1:45, 16:44]	0.874
LI‐HIIT	4:43 [3:10, 5:56]	
SI‐HIIT	6:04 [0:14, 12:17]	

*Note*: Time spent in each zone is presented as median [25th, 75th percentile]. % HR_max_, percentage of maximum heart rate measured during the cardiopulmonary exercise test. Bold values indicates *p* values < 0.05.

Abbreviations: LI‐HIIT, long interval high‐intensity interval training; SI‐HIIT, short interval high‐intensity interval training; VICT, vigorous intensity continuous training.

^a^ Post‐hoc test *p* < 0.05 versus VICT.

**FIGURE 3:**
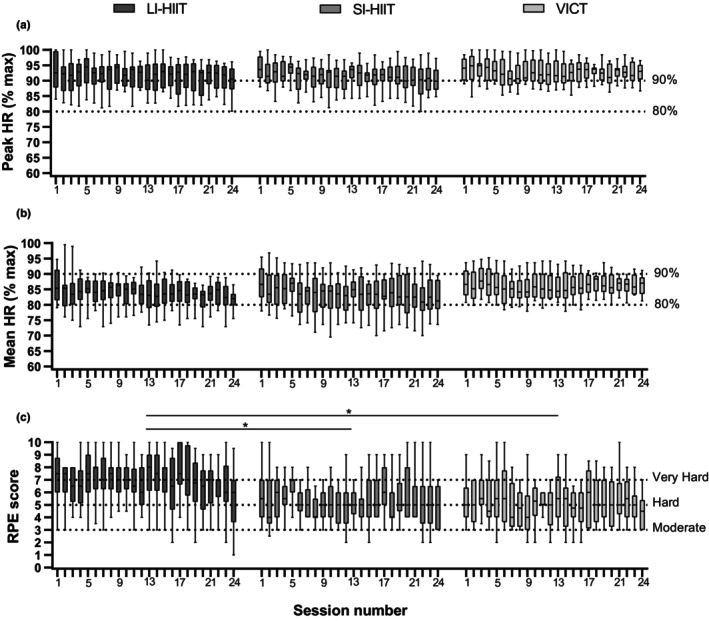
Boxplot of peak HR (a), mean HR (b), and RPE (c) during training sessions. HR, Heart Rate; LI‐HIIT, long interval high intensity interval training; RPE, rate of perceived exertion; SI‐HIIT, short interval high intensity interval training; VICT, vigorous intensity continuous training. *Post‐hoc test *p* < 0.05 between LI‐HIIT and other interventions.

As illustrated in Figure 3, a large inter‐subject variability was observed within each session for peak and mean HR, expressed as a percentage of HR_max_ measured during the CPET (A and B), and for RPE (C). Within each modality, between‐session variability was quite low for HR, with interquartile ranges for mean and peak HR staying respectively between 80%–90% and 90%–100% of HR_max_ throughout the intervention for the 3 modalities (Figure 3a,b), meaning the progression proposed ensured maintenance of mean and peak HR at similar levels all along the interventions. RPE interquartile ranges fluctuated between an effort perceived as moderate to very hard (Figure 3c).

Statistical analysis of pooled data from all sessions did not show a difference for mean HR (*p* = 0.28) and peak HR (*p* = 0.62) between the 3 training modalities. In contrast, the RPE median value was higher and the min to max range was larger during LI‐HIIT (median: 6.7, range: 3.6–8.2) compared to SI‐HIIT (median: 5.3, range: 3.9–7.0; *p* = 0.047) and VICT (median: 4.8 and range: 3.5–6.7; *p* = 0.025).

### 3.2 Body composition

Values of total and segmental body composition, and statistical analysis comparing the 3 groups at baseline and the effects of the interventions are presented in Table 3. No significant difference was found between groups at baseline. Total (+1%), leg (+1.5%), and trunk lean mass (+2%) were slightly increased (ANOVA time effect, all *p* < 0.01), while total and segmental fat mass did not change significantly after interventions (ANOVA time effect, all *p* > 0.05). Whatever the parameter, the statistical analysis did not reveal any significant intervention by time interaction (all *p* > 0.05).

**TABLE 3:** Baseline to post‐intervention comparison of the body composition components.

Parameters	Baseline	Post	Between groups comparison at baseline *p*‐value	ANOVA time *p*‐value/*η* ^2^ *p*	ANOVA time × group *p*‐value/*η* ^2^ *p*
Total body weight (kg)					
VICT	70.3 ± 12.3	71.0 ± 13.0	0.09	0.585/0.001	0.176/0.054
LI‐HIIT	77.9 ± 17.6	77.4 ± 18.5			
SI‐HIIT	65.9 ± 7.9	65.8 ± 7.7			
Total fat mass (kg)					
VICT	18.7 ± 10.6	18.8 ± 11.1	0.075	0.064/0.097	0.080/0.138
LI‐HIIT	25.9 ± 11.5	24.7 ± 11.4			
SI‐HIIT	18.0 ± 3.8	17.6 ± 3.8			
Total lean mass (kg)					
VICT	48.6 ± 8.2	49.3 ± 8.4	0.381	**0.002/0.255**	0.772/0.015
LI‐HIIT	49.9 ± 11.7	50.6 ± 11.9			
SI‐HIIT	44.8 ± 8.3	45.2 ± 8.1			
Leg fat mass (kg)					
VICT	8.1 ± 4.6	8.2 ± 4.6	0.110	0.078/0.089	0.112/0.121
LI‐HIIT	10.5 ± 3.5	10.1 ± 3.5			
SI‐HIIT	7.7 ± 1.9	7.6 ± 2.0			
Leg lean mass (kg)					
VICT	19.4 ± 3.2	19.5 ± 3.2	0.364	**0.002/0.261**	0.608/0.029
LI‐HIIT	19.9 ± 4.2	20.2 ± 4.4			
SI‐HIIT	17.9 ± 3.2	18.2 ± 3.2			
Arm fat mass (kg)					
VICT	1.8 ± 0.9	1.8 ± 0.9	0.060	0.543/0.011	0.313/0.066
LI‐HIIT	2.6 ± 1.1	2.5 ± 1.2			
SI‐HIIT	1.9 ± 0.5	1.9 ± 0.6			
Arm lean mass (kg)					
VICT	5.4 ± 1.5	5.3 ± 1.2	0.651	0.288/0.033	0.556/0.034
LI‐HIIT	5.5 ± 1.8	5.5 ± 1.9			
SI‐HIIT	5.0 ± 1.3	5.0 ± 1.3			
Trunk fat mass (kg)					
VICT	6.3 [4.2, 8.5]	6.2 [4.6, 7.8]	0.051	N/A	N/A
LI‐HIIT	9.2 [6.7, 14.9]	9.1 [6.1, 15.4]			
SI‐HIIT	6.9 [6.0, 9.6]	6.8 [6.3, 9.60]			
Trunk lean mass (kg)					
VICT	20.7 ± 3.8	21.2 ± 4.1	0.375	**0.007/0.194**	0.300/0.068
LI‐HIIT	21.3 ± 5.4	21.7 ± 5.3			
SI‐HIIT	18.9 ± 3.5	19.0 ± 3.4			

*Note*: Data presented as mean ± standard deviation or median [25th, 75th percentile] depending on distribution. Bold values indicates *p* values < 0.05.

Abbreviations: LI‐HIIT, long interval high intensity interval training; N/A, not applicable; *η* ^2^ *p*, partial eta‐squared; SI‐HIIT, short interval high intensity interval training; VICT, vigorous intensity continuous training.

### 3.3 Cardiorespiratory fitness

Values of the cardiorespiratory parameters and statistical analysis comparing the 3 groups at baseline, and the effects of the interventions are presented in Table 4. At baseline, there were no significant differences between groups for any of the parameters (all *p* > 0.05). The interventions improved VO_2_peak (+14% when expressed in L/min and in mL/min/kg of body weight, and +13% in mL/min/kg of lean mass), VT1 (+38% in L/min, +33% in mL/min/kg of body weight, and +36% in mL/min/kg of lean mass), and W_max_ (+16%; ANOVA time effect, all *p* < 0.001), while HR_max_ remained unchanged (ANOVA time effect, *p* = 0.26). No significant intervention by time interaction was found for any of the parameters (all *p* > 0.05).

**TABLE 4:** Baseline to post‐intervention comparison of the maximal cardiopulmonary exercise test parameters.

Parameters	Baseline	Post	Between groups comparison at baseline *p*‐value	ANOVA time *p*‐value/η^2^p	ANOVA time × group *p*‐value/η^2^p
VO_2_peak (L/min)					
VICT	2.73 ± 0.70	3.14 ± 0.72	0.497	***p* < 0.001/0.682**	0.25/0.077
LI‐HIIT	2.76 ± 0.76	3.01 ± 0.81			
SI‐HIIT	2.46 ± 0.63	2.81 ± 0.59			
VO_2_peak/LM (mL/kg/min)					
VICT	55.7 ± 7.5	63.3 ± 6.1	0.941	***p* < 0.001/0.664**	0.16/0.102
LI‐HIIT	55.2 ± 6.3	59.4 ± 7.2			
SI‐HIIT	54.7 ± 8.5	62.2 ± 7.2			
VO_2_peak/BW (mL/kg/min)					
VICT	39.3 ± 10.0	44.7 ± 9.6	0.815	***p* < 0.001/0.628**	0.44/0.047
LI‐HIIT	35.6 ± 6.5	39.3 ± 7.7			
SI‐HIIT	37.2 ± 7.7	42.7 ± 7.1			
W_max_ (Watts)					
VICT	223 ± 59	264 ± 69	0.445	***p* < 0.001/0.800**	0.24/0.082
LI‐HIIT	219 ± 52	248 ± 66			
SI‐HIIT	197 ± 48	230 ± 56			
HR_max_ (bpm)					
VICT	190 ± 10	189 ± 8	0.805	0.261/0.037	0.28/0.072
LI‐HIIT	189 ± 9	185 ± 11			
SI‐HIIT	192 ± 10	193 ± 10			
VT1 (L/min)					
VICT	1.38 ± 0.36	1.86 ± 0.53	0.549	***p* < 0.001/0.733**	0.80/0.013
LI‐HIIT	1.54 ± 0.51	1.95 ± 0.61			
SI‐HIIT	1.39 ± 0.35	1.84 ± 0.49			
VT1/LM (mL/kg/min)					
VICT	28.5 ± 5.6	37.6 ± 6.5	0.54	***p* < 0.001/0.752**	0.51/0.039
LI‐HIIT	30.9 ± 7.7	38.2 ± 7.3			
SI‐HIIT	30.8 ± 4.5	40.2 ± 5.6			
VT1/BW (mL/kg/min)					
VICT	20.0 ± 5.4	26.8 ± 7.6	0.85	***p* < 0.001/0.738**	0.57/0.033
LI‐HIIT	19.9 ± 5.5	25.2 ± 5.6			
SI‐HIIT	20.9 ± 3.7	27.6 ± 5.0			

*Note*: Data presented as mean ± standard deviation. W_max_ maximal output. Bold values indicates *p* values < 0.05.

Abbreviations: BW, body weight; LI‐HIIT, long interval high intensity interval training; HR_max_, maximal heart rate; LM, total lean mass; *η* ^2^ *p*, partial eta‐squared; SI‐HIIT, short interval high intensity interval training; VICT, vigorous intensity continuous training; VT1, first ventilatory threshold.

### 3.4 Neuromuscular parameters

Values of the neuromuscular parameters and statistical analysis comparing the 3 groups at baseline, and the effects of the interventions are presented in Table 5. There was no significant difference between groups at baseline for any of the parameters (all *p* > 0.05). After the interventions, an increase in endurance time (+12%) and MVC torque was observed (+3%), associated with a higher voluntary activation (+2%) and agonists aEMG (+10%), while biceps femoris aEMG decreased (−12%; ANOVA time effect, all *p* < 0.05). Resting twitch torque did not change significantly (*p* = 0.108). No significant intervention by time interaction was found for any of the parameters (all *p* > 0.05).

**TABLE 5:** Baseline to post‐intervention comparison of the neuromuscular parameters.

Parameters	Baseline	Post	Between groups comparison at baseline *p*‐value	ANOVA time *p*‐value/*η* ^2^ *p*	ANOVA time × group *p*‐value/*η* ^2^ *p*
Endurance time (s)					
VICT	138 ± 52	153 ± 68	0.168	**0.010/0.186**	0.462/0.046
LI‐HIIT	131 ± 40	152 ± 29			
SI‐HIIT	142 ± 54	148 ± 53			
MVC torque (N.m)					
VICT	199 ± 57	201 ± 58	0.339	**0.049/0.109**	0.295/0.069
LI‐HIIT	175 ± 49	177 ± 45			
SI‐HIIT	168 ± 51	179 ± 58			
Voluntary activation (%)					
VICT	93 ± 4	94 ± 3	0.535	**<0.001/0.283**	0.858/0.009
LI‐HIIT	90 ± 9	91 ± 7			
SI‐HIIT	91 ± 8	93 ± 6			
Agonists aEMG (μV)					
VICT	472 ± 164	493 ± 165	0.142	**0.012/0.172**	0.506/0.039
LI‐HIIT	350 ± 155	406 ± 193			
SI‐HIIT	469 ± 185	495 ± 196			
BF aEMG (μV)					
VICT	97 ± 43	79 ± 37	0.421	**0.026/0.142**	0.431/0.050
LI‐HIIT	91 ± 25	84 ± 29			
SI‐HIIT	81 ± 21	76 ± 25			
Twitch torque (N.m)					
VICT	76 ± 18	75 ± 18	0.455	0.108/0.074	0.644/0.026
LI‐HIIT	71 ± 21	70 ± 19			
SI‐HIIT	66 ± 18	66 ± 16			

*Note*: Data presented as mean ± standard deviation. Agonists aEMG, mean value of the average rectified electromyographic activities of the rectus femoris, vastus lateralis and vastus medialis during MVC. Bold values indicates *p* values < 0.05.

Abbreviations: BF aEMG, average rectified electromyographic activity of the biceps femoris during MVC; LI‐HIIT, long interval high intensity interval training; MVC, maximal voluntary contraction; *η* ^2^ *p*, partial eta‐squared; SI‐HIIT, short interval high intensity interval training; VICT, vigorous intensity continuous training.

The percentage of change in MVC torque was positively correlated to the percentage of change in voluntary activation (*r*_s_ = 0.40, *p* = 0.015) and agonists aEMG (*r*_p_ = 0.52, *p* = 0.001). The change in voluntary activation was negatively correlated with the baseline level of voluntary activation (*r*_s_ = −0.64; *p* < 0.001).

## 4 DISCUSSION

Since lack of time is the main perceived barrier to physical activity and higher intensity interventions are known to be more efficient in improving physical fitness, the aim of the present study was to compare the effectiveness of 3 high intensity cycling trainings conceived to fit easily between other daily activities. Cardiorespiratory fitness and muscle endurance were improved after the interventions and were accompanied by a small increase in lean mass and knee extensors isometric strength. Mean and peak HR during sessions, and time spent in the different intensity zones (60%–70%, 70%–80%, and above 90% of HR_max_) were not significantly different between the 3 modalities, except for a shorter time spent between 80% and 90% of HR_max_ during LI‐HIIT compared to VICT. In contrast, despite the shorter session duration and time spent between 80% and 90% of HR_max_, RPE was higher during LI‐HIIT compared to both SI‐HIIT and VICT.

### 4.1 Body composition

The absence of change in fat mass after the exercise intervention is in line with the literature on normo‐weighted subjects (Amatori et al., 2023). The increase in lean mass observed in the present study is small, and just above the coefficient of variation for lean mass measurement by DXA (~1%; Toombs et al., 2012). Its magnitude and location (leg and trunk) are, however, consistent with the lean mass increase reported by several studies after different cycling HIIT (Boutcher et al., 2019; Caparrós‐Manosalva et al., 2023; Gillen et al., 2013; Heydari et al., 2012; Trapp et al., 2008). The predominant effect on the leg and trunk lean mass is most probably related to the main role of leg and trunk muscles in cycling and as stabilizers, respectively (Trapp et al., 2008). To our best knowledge, the effects of LI‐HIIT, SI‐HIIT, and VICT on body composition were never directly compared. A potential explanation for the absence of difference between the 3 modalities could lie in a similar exercise‐induced metabolic stress, known to upregulate signaling pathways responsible for muscle hypertrophy (Callahan et al., 2021; Wackerhage et al., 2019).

### 4.2 Cardiorespiratory fitness

The statistical analysis revealed no difference between the 3 modalities regarding the improvement of cardiorespiratory fitness (VO_2_peak, W_max_, and VT1). Our results are consistent with a recent meta‐analysis showing that LI‐HIIT and SI‐HIIT/SIT were equally effective to improve cardiorespiratory fitness (de Oliveira‐Nunes et al., 2021). However, the authors did not compare the effectiveness of SI‐HIIT (using near maximal or slightly supramaximal intensities) with Wingate‐based or all‐out versions of SIT. To our knowledge, solely Bayati et al. (2011) assessed cardiorespiratory adaptations induced by a Wingate‐based SIT and a SI‐HIIT as in the present study (i.e., 30‐s intervals at 125%W_max_ interspaced with 2 min of recovery). They reported that both modalities similarly improved cardiorespiratory fitness. This observation and present results support the use of SI‐HIIT, as it appears to induce comparable gains to all‐out SIT and is more adapted for the general population.

HIIT and SIT were shown to be equally effective for improving cardiorespiratory fitness (de Oliveira‐Nunes et al., 2021) and to be superior to MICT (Sultana et al., 2019). Since cardiorespiratory fitness improvements depend on training intensity (MacInnis & Gibala, 2017), and more specifically on the time spent at near maximal intensity (Buchheit & Laursen, 2013a; Midgley & Mc Naughton, 2006), conclusions drawn from MICT cannot be extrapolated to VICT. The current findings demonstrate a comparable increase in VO_2_peak, W_max_, and VT1 after the 3 training modalities, likely due to the similar time spent above 90% of HR_max_. Consequently, VICT appears to be as effective as LI‐HIIT and SI‐HIIT in enhancing cardiorespiratory fitness.

### 4.3 Knee extensors torque and voluntary activation

Unlike cardiorespiratory and body composition adaptations, neuromuscular adaptations to different HIIT were barely investigated, and our study is the first to assess MVC torque and voluntary activation changes in response to different HIIT and a VICT. After cycling HIIT interventions in inactive or recreationally active subjects, a 7% increase in the MVC torque of the knee extensors was observed by Martinez‐Valdes et al. (2017) after only 6 sessions of SI‐HIIT, Caparrós‐Manosalva et al. (2023) reported a 10% increase after a 12‐week SI‐HIIT (3 times/week), and we observed a 3% increase after our 8‐week HIIT and VICT interventions. In contrast, Bruseghini et al. (2019) and Lewis et al. (2017) did not find any change after 8 weeks of LI‐HIIT (3 times/week) and 6 sessions of SIT, respectively. These inconsistent isometric strength improvements in response to HIIT interventions are certainly related to the diversity of training protocols (duration, modality, etc.) and the suboptimal stimulus induced by HIIT to increase muscle strength compared to resistance training (Carvalho et al., 2022). However, differences between subjects' initial voluntary activation level, and thus ability to reach their maximal torque, could also partly explain these discrepancies. This is supported by present results and by our previous study on whole‐body HIIT (Scoubeau et al., 2023), which shows a positive correlation between the small changes in MVC torque and voluntary activation and a negative correlation between baseline voluntary activation and its change after intervention, suggesting a neural contribution to the torque increase that is more pronounced in subjects with a lower activation level at baseline. In contrast, resting twitch torque did not change, suggesting there was no change in muscle contractile properties after our interventions (Alway et al., 1989).

The absence of a significant difference between the 3 interventions could be related to the small increase in MVC torque, but also to the fact that, besides the absolute loads attained during training sessions, total mechanical work also influences the extent of motor unit recruitment and strength improvement (Alegre et al., 2015). During VICT, maximal instantaneous training loads are lower compared to LI‐HIIT and SI‐HIIT. However, the constant load (~70% VO_2_peak) is sustained during a longer period, leading to a consequent total mechanical work and recruitment of both type I and II motor units, as previously reported during cycling exercises of comparable intensities (Altenburg et al., 2007; Gollnick et al., 1974).

Although the small strength improvement observed in present and previous studies is encouraging, its functional significance in healthy subjects and other populations must be confirmed by further studies. The latter should also investigate if longer training periods could potentiate these strength improvements.

### 4.4 Muscle endurance

An increase in muscle endurance, quantified by the endurance time during a submaximal isometric contraction, was previously reported after whole‐body HIIT (Scoubeau et al., 2023) and cycling MICT (Martinez‐Valdes et al., 2017; Vila‐Chã et al., 2010). The present study shows that muscle endurance is also improved after 8 weeks of HIIT and VICT, with no significant difference between training modalities. Mechanisms that could explain the enhanced muscle endurance after the 3 high‐intensity trainings are an increased resistance to inhibitory actions of III/IV muscle afferents and muscle pain tolerance, improvements in the muscle mitochondrial content and function, and buffering capacity reported after high‐intensity exercise interventions (Bishop et al., 2019).

### 4.5 Perceived exertion and intensity during training sessions

As previously reported during prolonged constant load exercise (Mikus et al., 2009; Souissi et al., 2021), cardiovascular drift (i.e., progressive increase in HR) occurred during VICT, which explains that mean and peak HR and time spent >90% of HR_max_ were comparable to LI‐HIIT and SI‐HIIT. Surprisingly, we did not observe a higher RPE for VICT compared to SI‐HIIT as previously reported by Jung et al. (2014). This difference is most probably related to the higher intensity of their VICT (80% of W_max_) than in the present study (70% of VO_2_peak).

Although the session duration and the time spent between 80% and 90% of HR_max_ during LI‐HIIT were shorter, RPE during sessions was higher throughout the intervention compared to VICT and SI‐HIIT. A higher RPE was previously reported during single sessions of LI‐HIIT compared to SI‐HIIT and moderate to vigorous intensity continuous training (Bartlett et al., 2011; Kilpatrick et al., 2015; Naves et al., 2019; Oliveira et al., 2013), but to our knowledge it was never investigated during interventions of a few weeks.

Perceived exertion was defined by Marcora as “the conscious sensation of how hard, heavy, and strenuous a physical task is” (Marcora & Staiano, 2010). It is generated by neural process of the efferent copies from the central motor command and respiratory drive, and is indirectly modulated by afferent feedback from muscles and cardiorespiratory system (Lopes et al., 2022; Pageaux, 2016). Compared to VICT or SI‐HIIT, LI‐HIIT is associated with longer bouts at intense workload, inducing longer periods of elevated ventilation, HR, higher muscle recruitment, and accumulation of fatigue related metabolites (Allen et al., 2008). Therefore, central motor command and group III/IV afferents stimulation should be greater during LI‐HIIT and increase the perceived exertion.

### 4.6 Implications

Considering the potential negative effect of elevated RPE on the affective response to exercise (Farias‐Junior et al., 2020), one should keep in mind these differences in RPE when prescribing exercises, especially for individuals unaccustomed to regular physical activity (Lopes et al., 2021). Altogether, our results suggest that it is advisable to start with SI‐HIIT and/or VICT at ~70% VO_2_peak. LI‐HIIT could be introduced once subjects' capacity to cope with the physical and cognitive demands of exercising at higher intensities is improved (Kilpatrick et al., 2015), especially if time is a constraint, as shorter sessions of LI‐HIIT appear to induce comparable cardiorespiratory, lean mass, and neuromuscular adaptations to SI‐HIIT and VICT.

### 4.7 Limitations

In the present study, the necessity to fit the training sessions within a ~30 min time‐window with achievable targets for mostly inactive subjects made the volume equalization between groups impractical. Some argue this could limit the inter‐group comparison in terms of physiological adaptations (Stern, 2022). However, as mentioned by Vollaard et al. (2023), it is not always possible and justified to equalize volume/energy expenditure without running the risk of comparing protocols that are unlikely to work or not achievable for the target population. Another limitation that could be raised is the absence of monitoring of dietary and physical activity habits between exercise sessions. However, our subjects were reminded not to modify their physical activity and dietary habits. Regarding dietary habits, recording their diet may alter food intake, and bias related to self‐report could happen unintentionally or to reduce burden (Shim et al., 2014). We therefore chose not to record dietary intakes. Also, it must be mentioned that the LI‐HIIT group had a significantly higher BMI (see Table 1) and a tendency for higher fat mass at baseline. However, since fat mass remained unchanged and other parameters showed similar changes after the three interventions, this initial difference in BMI likely had a minimal impact on the overall outcomes. Lastly, our sample was relatively small and composed of inactive to moderately active healthy subjects, and the intervention was limited to 8 weeks. This emphasizes the need for further research to provide additional insights into the effects on health‐related fitness, in larger and more diverse populations, and to investigate the risk/benefit ratio and long‐term effects of these training modalities.

## 5 CONCLUSIONS

VICT, SI‐HIIT, and LI‐HIIT improved cardiorespiratory fitness and muscle endurance, with a modest increase in MVC torque, voluntary activation, and lean mass. Despite a shorter session duration for LI‐HIIT, the similar time spent at near maximal intensity during the 3 interventions probably explains the lack of significant differences in training‐related improvements. Based on the assessment of RPE during training sessions, SI‐HIIT and VICT were perceived as less strenuous than LI‐HIIT, suggesting they could be better tolerated by subjects unaccustomed to vigorous exercise. In this context, proposing SI‐HIIT, VICT, and LI‐HIIT stepwise, or as complementary options, based on previous experience of physical activity, preferences, and time constraints could positively influence affective response to exercise, increase intervention diversity, and thereby promote motivation and adherence.

## FUNDING INFORMATION

This study was funded by the Brussels Region (INNOVIRIS BRIDGE; grant DiaType). This paper is published with the support of the “Fondation Universitaire de Belgique” (Belgian University Foundation).

## CONFLICT OF INTEREST STATEMENT

The authors declare no conflict of interest.

## ETHICS STATEMENT

Ethics approval was provided by the the Erasmus hospital (Brussels, Belgium) Ethical Committee (reference: B406201836213).

## Data Availability

All the data supporting the conclusions of the present study will be made available from the corresponding author upon reasonable request.
